# Endogenous Small RNA Mediates Meiotic Silencing of a Novel DNA Transposon

**DOI:** 10.1534/g3.115.017921

**Published:** 2015-06-23

**Authors:** Yizhou Wang, Kristina M. Smith, John W. Taylor, Michael Freitag, Jason E. Stajich

**Affiliations:** * Department of Plant Pathology & Microbiology and Center for Plant Cell Biology, Institute for Integrative Genome Biology, University of California-Riverside, Riverside, California 92521; † Plant Sciences Graduate Program, University of California, Riverside, California 92521; ‡ Department of Biochemistry and Biophysics, Oregon State University, Corvallis, Oregon 97331; § Department of Plant and Microbial Biology, University of California, Berkeley, California 94720

**Keywords:** meiotic silencing, small RNA, transposon, genome defense, *Neurospora*

## Abstract

Genome defense likely evolved to curtail the spread of transposable elements and invading viruses. A combination of effective defense mechanisms has been shown to limit colonization of the *Neurospora crassa* genome by transposable elements. A novel DNA transposon named *Sly1-1* was discovered in the genome of the most widely used laboratory “wild-type” strain FGSC 2489 (OR74A). Meiotic silencing by unpaired DNA, also simply called meiotic silencing, prevents the expression of regions of the genome that are unpaired during karyogamy. This mechanism is posttranscriptional and is proposed to involve the production of small RNA, so-called masiRNAs, by proteins homologous to those involved in RNA interference−silencing pathways in animals, fungi, and plants. Here, we demonstrate production of small RNAs when *Sly1-1* was unpaired in a cross between two wild-type strains. These small RNAs are dependent on SAD-1, an RNA-dependent RNA polymerase necessary for meiotic silencing. We present the first case of endogenously produced masiRNA from a novel *N. crassa* DNA transposable element.

Genome protection is of paramount importance during sexual reproduction when DNA is replicated for packaging into gametes because, at this time, it is particularly susceptible to invasion by viruses and transposable elements (TEs) (Calvi and Gelbart 1994; Collins *et al.* 1987; Pelisson *et al.* 2002; Prudhomme *et al.* 2005; Shapiro 2005). To protect host genomes from attack by foreign elements, “genome defense” mechanisms have evolved that use gene silencing or targeted mutations to combat nonself elements (Aravin *et al.* 2003; Goodier and Kazazian 2008; Lau 2010). The fungus *Neurospora crassa* has at least three genome defense mechanisms that have limited the colonization of its genome by selfish elements (Galagan *et al.* 2003). The genome defense systems include the irreversible repeat-induced point mutation (RIP) (Selker and Garrett 1988; Cambareri *et al.* 1989) and two reversible posttranscriptional mechanisms, the RNA interference (RNAi)-like “quelling” (Romano and Macino 1992; Cogoni *et al.* 1994) and meiotic silencing (Aramayo and Metzenberg 1996; Shiu *et al.* 2001; Shiu and Metzenberg 2002). RIP is a premeiotic hypermutation process that targets duplicated segments of DNA (Selker and Garrett 1988; Cambareri *et al.* 1989) by converting C:G to T:A in both copies of the duplicated regions. Quelling is a posttranscriptional, small RNA-based gene-silencing pathway that has so far been only studied in detail in the asexual stages of the life cycle (Fulci and Macino 2007). The third genome defense system, first considered a form of “transvection” (Aramayo and Metzenberg 1996) and later called meiotic silencing by unpaired DNA (MSUD) (Shiu and Metzenberg 2002; Shiu *et al.* 2001) or simply meiotic silencing (Kelly and Aramayo 2007), occurs after karyogamy and targets transcripts that originate from regions with dissimilar DNA sequence and are therefore are unpaired. The system also affects RNA that is produced from additional paired alleles (Aramayo and Metzenberg 1996; Shiu *et al.* 2001).

The mechanism for detection of unpaired regions remains elusive, although DNA repair components have been linked to its efficiency (Samarajeewa *et al.* 2014). Genetic crosses of strains with unpaired regions show transient silencing of transcripts from genes in these region (Shiu *et al.* 2001; Lee *et al.* 2004; Shiu *et al.* 2006; Alexander *et al.* 2008), and this silencing is limited to stages from early karyogamy until ascospore, as tracked by expression of histone H1-green fluorescence protein fusion genes (Jacobson *et al.* 2008). It is hypothesized that RNAs produced from unpaired regions are detected as “aberrant” and subject to RNAi-mediated silencing (Lee *et al.* 2004). Many mutated genes affecting meiotic silencing are homologous to genes in RNAi pathways in plants, fungi, and animals. These genes include *sad-1*, a putative RNA-dependent RNA polymerase (Shiu and Metzenberg 2002; Shiu *et al.* 2001), *dcl-1/sms-3*, a Dicer-type exonuclease (Alexander *et al.* 2008), *sms-2*, an Argonaute homolog (Lee *et al.* 2003), QIP, which converts duplex RNA into siRNAS (Xiao *et al.* 2010; Lee *et al.* 2010a), and additional scaffold proteins and components SAD-2, SAD-3, SAD-4, SAD-5, and SAD-6 (Xiao *et al.* 2010; Hammond *et al.* 2011, 2013b; Samarajeewa *et al.* 2014; Decker *et al.* 2015). Suppression of meiotic silencing in some cases has enabled meiotic drive elements such as Spore killer (Raju *et al.* 2007; Hammond *et al.* 2012; Harvey *et al.* 2014). Recent work in support of the hypothesis that RNAi is involved in meiotic silencing used an engineered deletion at the *Rsp* locus to show that small RNAs are produced from this unpaired region during meiosis (Hammond *et al.* 2013a). However, small RNAs have not yet been reported from matings between wild-type strains with unpaired regions segregating in natural populations.

TEs present in only one parent will be unpaired during sexual crosses and thus become natural substrates for meiotic silencing. One proposed role for meiotic silencing and other genome defense mechanisms has been to control the spread of TEs (Nolan *et al.* 2005; Catalanotto *et al.* 2006; Girard and Hannon 2008). So far, however, there has been no direct demonstration for a role of this genome defense system in the control of TEs. This lack can be explained, in part, by the few active TEs in *N. crassa*. Currently only *Tad* (Transposon in Adiopodumé), a long interspersed element−like retroelement found intact and active in the Adiopodumé strain, has been demonstrated to transpose (Kinsey 1989; Kinsey and Helber 1989; Kinsey *et al.* 1994; Zhou *et al.* 2001). In addition, relics of TEs that have accumulated as a consequence of RIP have been described in the *N. crassa* reference genome derived exclusively from FGSC 2489 (OR74A) (Selker *et al.* 2003).

By comparing genomes of several laboratory strains, multiple loci in the reference 2489 from the Fungal Genetics Stock Center (FGSC, University of Missouri, Kansas City, MO) were identified to be missing among individuals in this pedigree. One of the largest of these detected insertion/deletions is a TE we named *Sly1-1*. When we sequenced small RNAs from three stages during premeiotic and meiotic development, we detected “meiotic-silencing-associated small interfering RNAs” (masiRNAs) that originated from *Sly1-1*. Here, we present evidence that *Sly1-1* is an active DNA-type transposon that is recognized by meiotic silencing when unpaired during meiosis. Furthermore, we confirm that the meiotic silencing machinery is required for the production of masiRNAs emanating from *Sly1-1*. Thus we provide support for the role of meiotic silencing in genome defense through detection of meiotic small RNAs targeting a TE in an unpaired state.

## Materials and Methods

### Strains and growth conditions

Neurospora strains used in this study are listed in Supporting Information, Table S1. All strains were originally obtained from the FGSC and are maintained in the senior author’s laboratories. Mycelia of strains used for DNA extraction were collected after growth in liquid Vogel’s medium N (Vogel 1956) at 25° for 3 d. Vegetative tissues of FGSC 2489 for RNA extraction was collected after growth on solid Vogel’s medium N in the dark at 30° for 3 d, followed by growth in the light at room temperature for 2 d. For tissue collection during sexual development, FGSC 2489 was first grown on synthetic crossing medium (Westergaard and Mitchell 1947) covered with cellophane (Midsci, St. Louis, MO) in 245 × 245 × 25-mm bioassay dishes (Thermo Scientific, Hvidovre, Denmark). After 10 d of growth, at room temperature in light/dark conditions, we looked for protoperithecia (PP) under a dissecting microscope, and then harvested tissues enriched with PP by scraping from the cellophane, followed by flash-freezing in liquid nitrogen. Additional plates of PP were crossed with either the wild-type FGSC 8820 or *Sad-1^Δ^* strains. Regions enriched with perithecia were cut by sterile razor blades after 2, 4, and 6 d postfertilization (PF). Tissues from these regions were scraped from cellophane and flash-frozen in liquid nitrogen. All collected tissues were stored at −80° until further use.

### RNA extraction and small RNA Northern blots

Harvested sexual tissue was processed for RNA extracted for northern blots or small RNA sequencing at least four separate times during the project each from new crosses of the FGSC 2489 and FGSC 8820 strains. Tissues were ground by mortar and pestle in a liquid nitrogen bath and transferred to a Falcon tube. Ground tissues were homogenized with at least 1 mL of Trizol reagent (Ambion, Carlsbad, CA) per 50−100 mg of tissue and vortexed thoroughly. To each 1 mL of Trizol, 200 μL of chloroform was added, vortexed for 15–30 sec, and incubated at room temperature for 5 min. Samples were centrifuged at 13,000*g* for 15 min at 4°, the aqueous phase was transferred to a clean tube, and 500 μL of isopropanol for each 1 mL of Trizol was added. Precipitated RNA formed a compacted pellet after centrifuging at 13,000*g* for 20 min at 4° and the supernatant was removed. The pellet was washed with 80% ethanol, vortexed, centrifuged at 7500*g* for 5 min, and allowed to air dry for 10 min. Total RNA solution was obtained after dissolving the pellet in diethylpyrocarbonate-treated water. Polyethylene glycol8000 and NaCl were added into total RNA with final concentration 5% polyethylene glycol and 0.5 M NaCl to differentially precipitated high-molecular-weight RNAs followed by sitting on ice for 2 hr. Low-molecular-weight RNAs were recovered from the supernatant by ethanol precipitation, resolved by diethylpyrocarbonate-treated water and quantified in a NanoDrop 2000c spectrophotometer (Thermo Scientific, Waltham, WA). Approximately 10 μg of isolated low-molecular- RNAs were separated on a 15% denaturing polyacrylamide-urea gel in with a miRNA marker was used as molecular mass standard (New England Biolabs, Ipswich, MA). RNA was transferred to a Hybond-NX membrane (Amersham Biosciences, Freiburg, Germany) in 0.5× TBE using Trans-Blot Electrophoretic Transfer Cell apparatus (Bio-Rad, Hercules, CA) at 14V overnight. Constitutively expressed 18S ribosomal RNA was used as a control to test for equal loading of RNA by staining membranes with ethidium bromide for visualization. Carbodiimide-mediated crosslinking for 2 hr at 60° was used to crosslink RNA to Hybond-NX membranes followed by baking at 80° for 1 hr (Pall *et al.* 2007).

Twelve primer pairs were used to amplify the NCU09969 locus (Table S4). The polymerase chain reaction (PCR) products were ∼500 bp and arranged end to end. Each of the amplicons were verified by sequencing and mixed together as templates to make ^32^P-labeled DNA probes. Prehybridization and hybridization was performed in PerfectHyb Plus hybridization buffer (Sigma-Aldrich, St. Louis, MO), at 42° overnight. To remove unspecific background, the membrane was washed twice in 2× saline sodium citrate (SSC; 0.3M NaCl, 30mM sodium citrate, pH 7.0) and 0.1% sodium dodecyl sulfate (SDS) at 40° for 15 min, and once in 0.5× SSC (75 mM NaCl, 7.5 mM sodium citrate, pH 7.0) and 0.1% SDS at 40° for 15 min. Finally, the membranes were exposed to a phosphoimager screen and scanned after 24 hr on a Typhoon 941 phosphoimager. Results were analyzed by Image Quant TL (version 7.0) software.

### DNA extraction and Southern blots

Mycelia from strains were grown in liquid culture, filtered on Whatman paper, and air dried in a Buchner funnel by vacuum suction and weighed. Approximately 1 mg of mycelium of each strain was ground by mortar and pestle in a liquid nitrogen bath and homogenized with 600 μL of cell lysis buffer (QIAGEN, Valencia, CA) and 3 μL of Proteinase K (QIAGEN) at 60° for 1−2 hr. After cooling to room temperature, 200 μL protein precipitation solution (QIAGEN) was added to each sample and the solution kept on ice for 10 min. Samples were centrifuged at 13,000*g* for 15 min and 500 μL of clear supernatant was transferred to a new tube followed by adding 500 μL of isopropanol for DNA precipitation. Samples were centrifuged at 13,000*g* for 10 min and a compacted DNA pellet formed, which was washed with 1 mL of cold 70% ethanol, followed by air-drying and resuspension in sterile water.

The restriction enzymes *Xho*I, *Afl*III, *Nco*l, *Dra*III, and *Sty*l were used separately to digest ∼20 μg of genomic DNA from various strains. Digested products were separated in 0.8% agarose gels, followed by blotting onto nylon membranes (GE Healthcare Bio-Sciences, Pittsburgh, PA). Four PCR products were used as templates to synthesize four ^32^P-labeled DNA probes (probes A, B, C, and D, respectively; Table S5). Probes A, B, and C were hybridized to blots with *Xho*I-digested DNA. Probe D was hybridized to blots with *Afl*III-, *Nco*l-, and *Dra*III-digested DNA. Prehybridization and hybridization was performed in PerfectHyb Plus hybridization buffer (Sigma, Deisenhofen, Germany) at 65° overnight. Membranes were washed one time at 65° in four solutions [2× SSC (0.3 M NaCl, 30 mM sodium citrate, pH 7.0) and 0.1% SDS, 1× SSC (0.15 M NaCl, 15 mM sodium citrate, pH 7.0) and 0.1% SDS, 0.5× SSC (75 mM NaCl, 7.5 mM sodium citrate, pH 7.0), and 10% SDS, 0.1× SSC (7.5 mM NaCl, 1.5 mM sodium citrate, pH 7.0)] and 0.1% SDS for 15 min, respectively, to minimize unspecific background. The membranes were exposed to a phosphoimager screen and scanned after 24 hr on a Typhoon 941 phosphorimager. Results were analyzed by Image Quant TL (version 7.0) software.

### Real-time quantitative (qRT)-PCR

Equal amounts (∼2 μg) of DNase I (Invitrogen, Carlsbad, CA) treated total RNAs were reverse transcribed with SuperScript II reverse transcriptase (Invitrogen) using random hexamers. The 10-μL qRT-PCR system was used, including ∼50 ng of cDNA, 10 μL of iQ SYBR Green Supermix (Bio-Rad, Hercules, CA), and 150 nM primers. The *N. crassa* β-tubulin gene (NCU04054) was used as an internal control for qRT-PCR. Each reaction was in triplicate and performed in a Bio-Rad CFX 96 Real-Time PCR machine. Primer sequences are listed in Table S5. Data analysis was performed using CFX Manager Software v3.1 to calculate the fold change using delta-delta Ct values. A sample from vegetative growth was used as control sample to calculate the relative RNA levels.

### Small RNA sequencing and FGSC 8820 genome sequencing

Total RNAs from samples of three time points (PP, 2d PF, 4d PF) were extracted with an miRNeasy Mini kit (QIAGEN). Small RNA sequencing library are constructed by following the standard protocols of Illumina TruSeq Small RNA Sample Prep kit in the University of Utah Sequencing Core. Size from 145 to 160 bp small RNA with adaptors (118 bp) were isolated and sequenced on an Illumina Genome Analyzer IIx to generate 50-nt single end reads. Sequence reads from three time points (PP, 2d PF, 4d PF) are deposited in the SRA database under project accession number SRP021051. Additional pilot sequencing of the 4d PF time point, which had been previously prepared in a similar fashion and sequenced on an Illumina Genome Analyzer at University of British Columbia Sequencing center serves as replicate for comparison, though with considerably lower sequencing coverage.

The genomic sequencing library of strain FGSC 8820 was constructed by using Nextera Illumina DNA preparation kit with dual indexing primers and sequenced in the Genomic Core at the Institute of Integrative Genome Biology, University of California, Riverside, on an Illumina HiSeq2000 genome analyzer. The sequence coverage was approximately 80X, and the reads are deposited in the SRA database under project accession SRP021049.

### Small RNA sequence analysis

**Figure 1:**
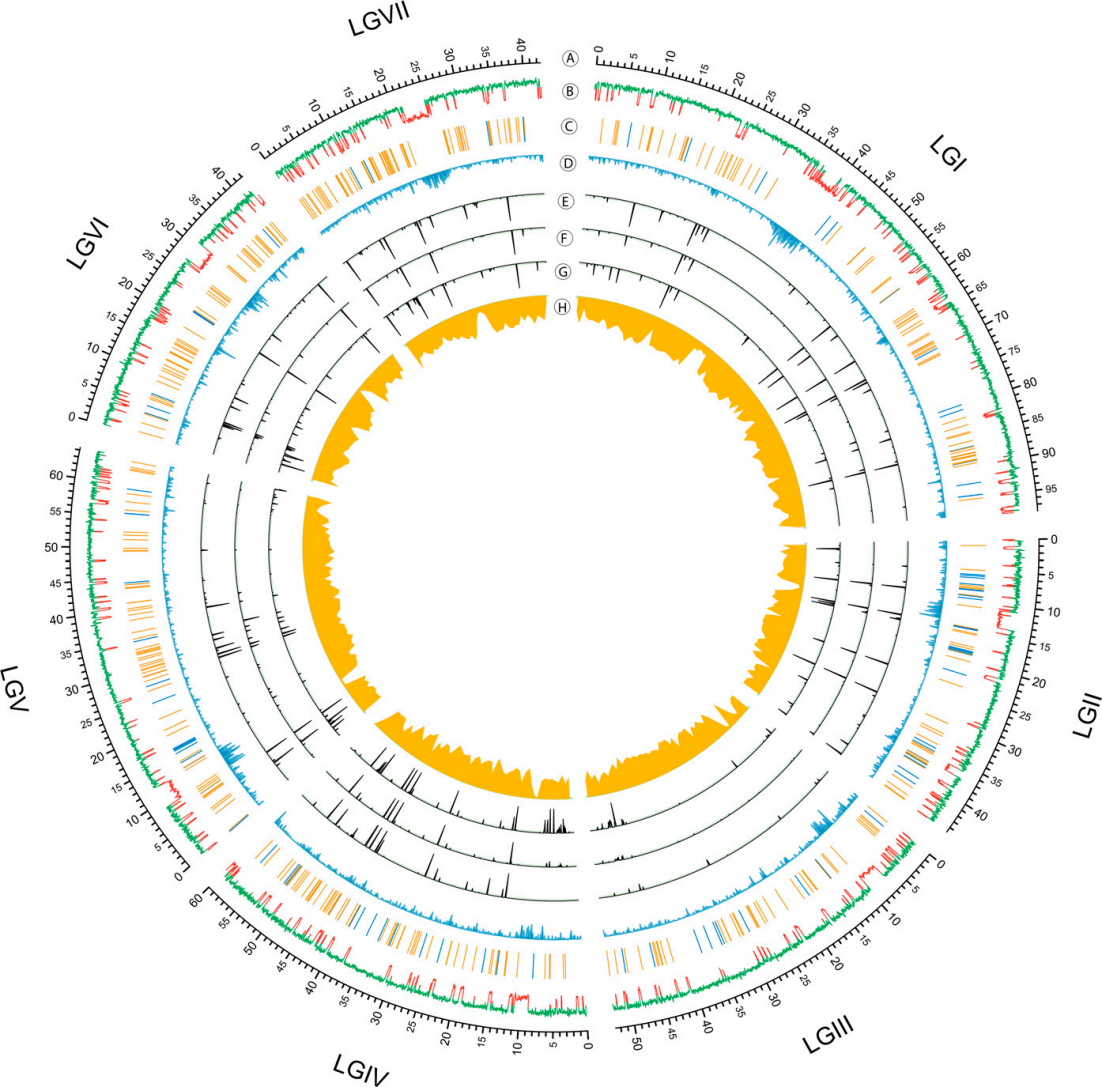
Circular genome visualization and data visualization with Circos. From outside in (A) seven linkage groups of *N. crassa*; (B) global profile of RIP shown by the composite repeat-induced point mutation (RIP) index [CRI; (Lewis *et al.* 2009)]; positive and negative CRI values imply that DNA has been subjected to RIP or not, presented by red and green on the plot, respectively; (C) rRNA (blue bar) and tRNA locus (orange bar); (D) repeat density based on a library of repeats from RepBase and a curated collection derived from multiple *Neurospora* species (Gioti *et al*., 2013); (E−G) small RNA sequence profiles from three time points in sexual development: protoperithecia of FGSC 2489, 2 d and 4 d postfertilization from a cross between FGSC 2489 and FGSC 8820; (H). Read depth of FGSC 8820 genome sequencing aligned to the FGSC 2489 genome.

Illumina sequence reads in FASTQ format were processed to remove low quality and artifactual reads, trim Illumina adapter sequences with the fastx_toolkit. (http://hannonlab.cshl.edu/fastx_toolkit/). Reads longer than 17 nt and smaller than 30 nt were mapped to the reference genome assembly (*N. crassa* version 12 – accession AABX03000000.3) by Bowtie v2.1.0 (Langmead and Salzberg 2012), allowing for no mismatches. SAM and BAM files were manipulated with SAMtools v1.1 (Li *et al.* 2009) and Picard v1.81 (http://broadinstitute.github.io/picard). Identification of reads aligning to unpaired regions was performed using BEDtools v2.17.0 (Quinlan and Hall 2010) and custom scripts written in Perl (v5.10.1) (http://github.com/stajichlab/neurospora_MSUD). An annotation file of noncoding RNAs was created by aligning the known sequences for ribosomal RNA (rRNA; accession FJ360521.1), small nucleolar RNAs (Liu *et al.* 2009) microRNA-like (milRNA) and Dicer-independent small interfering RNA loci [Table S2 and Table S3 from Lee *et al.* (2010b)] to Nc12 assembly with BLAT (Kent 2002). The transfer RNAs (tRNAs) were predicted with tRNAScan-SE (Lowe and Eddy 1997). Small RNAs that aligned to the mitochondrial genome were classified as mitochondrial RNA; other small RNAs that aligned to genome regions without any gene or repetitive element annotation were grouped into the “other” category. The genome representation as a Circos plot (Figure 1) was generated with Circos version 0.66 (Krzywinski *et al.* 2009); the configuration scripts are available at http://github.com/stajichlab/neurospora_MSUD. Figures displaying the distribution of smallRNA classes, read length, 5′ base preference, and strand specificity were made in R (http://r-project.org) and postprocessed with Adobe Illustrator.

### Analysis of resequencing data

Analysis of Illumina genome sequencing of strains FGSC 2489, FGSC 8820, and 19 classic mutants (McCluskey *et al.* 2011) was performed to test if *Sly1-1* could be detected in any other strains. FGSC 2489 sequencing data were taken from public SRA accessions (SRR018138-SRR018144). Chromatin immunoprecipitation sequencing of the NMF229 strain also was analyzed in the same fashion. Sequences were trimmed for quality with sickle (http://github.com/najoshi/sickle) requiring at least phred quality of 30 and length of 25 bp. Paired end data processing required both pairs were required to pass filtering and quality control or else the entire pair was discarded. Trimmed and filtered short read DNA sequences were aligned to the genome with BWA v0.7.9-r783 using ‘bwa aln –q 20,’ which was further processed to produce SAM and sorted BAM files with SAMtools and Picard. Multiple matches were allowed to support the presence of multiple *Sly-1* loci in the genome. Visualization of the read depth, and presence or absence of reads in the *Sly1-1* locus was performed in the Genome Browser and computed using BEDtools v2.17.0 (Quinlan and Hall 2010).

### Transposon bioinformatics analysis

The superfamily classification of *Sly1-1* was performed by searching the sequence against a curated library of DNA transposase domains (Yuan and Wessler 2011) to identify it as likely member of the CMC superfamily (CACTA – *Mirage* – *Chapev* and *Transib*). A multiple alignment was constructed of these homologs and the *Ch. globosum* and *Co. immitis* copies and a phylogenetic tree constructed with RAxML (v7.3.2) (Stamatakis 2006) using a BLOSUM62 model with empirical frequencies. Internal node support in the tree was estimated from 100 bootstrap replicates. Identification of the specific D-D-E residues was performed by predicting secondary structure of the protein using PSIPRED of the DDE domain (Buchan *et al.* 2013; Jones 1999) and identifying the residues falling within the correct range of expected folds (Yuan and Wessler 2011). The distance between the DDE triad and the range of signature motifs C(2)C and H(3)H are consistent with the distance observed in members of the CMC family.

### Terminal inverted repeat (TIR) and target-site duplication (TSD) analysis methods

Terminal inverted repeat 1 and 2 (TIR1 and TIR2) were identified by self-aligning putative regions of *Sly1-1* including its 3-kb flanking DNA sequences upstream and downstream, using NCBI-BLASTN (Altschul *et al.* 1997). We then detected clear breaks in reads adjacent to the upstream TIR1 and downstream TIR2 in strains from with *Sly1-1* is absent. These were considered breakpoints between TIRs and TSDs. In the strains with *Sly1-1* the reads located at breakpoints are continuous. Sequences of TSDs were determined according to these continuous reads by detecting their common sequences adjacent to the TIRs.

### Annotation of regions that have undergone RIP

Testing for copies of *Sly1-1* that have undergone RIP was done by RIPCAL (Hane and Oliver 2008) based on a multiple alignment of all homologs of the TE sequence. In addition, whole genome RIP analysis was performed with the script RIP_index_calculation.pl (http://github.com/hyphaltip/fungaltools) by computing RIP indices in sliding windows across all chromosomes. The RIP index for each locus was evaluated visually as a track in the Stajich lab Genome Browser (http://gb2.fungalgenomes.org).

### Data visualization

Genome sequence, annotation, and BAM files for aligned reads were loaded into the Generic Genome Browser (Stein *et al.* 2002), hosted by the Stajich lab at http://gb2.fungalgenomes.org for visualization.

### Data availability

Whole-genome and small RNA sequencing reads are deposited in the Short Read Archive under accession numbers: SRP021049 and SRP021051. Perl scripts and processed data files used in this project are available from http://github.com/stajichlab/neurospora_MSUD. Trimmed short reads and alignments are available at http://fungalgenomes.org/public/neurospora/data/support_files/Wang_MSUD.

## Results and Discussion

### Detection of small RNAs during the sexual cycle

To identify small RNAs produced from unpaired regions during meiotic silencing (masiRNAs; Hammond *et al.* 2013a), we crossed the laboratory wild-type strain, FGSC 2489, with FGSC 8820, a progeny from crosses of wild collected strains. We isolated pools of small RNA from tissues at three different times: before fertilization ( PP), 2d PF, and 4d PF. After trimming adaptor sequences and eliminating spurious RNAs or degradation products, we obtained abundant short read sequences from the samples (PP 22,538,276; 2d PF 21,562,565; 4d PF 22,731,295). These reads were mapped to the *N. crassa* reference genome assembly 12 (AABX00000000.3) to identify genomic origins of small RNAs. Analysis of read coverage indicated that some genomic regions were highly enriched in small RNA production, including subtelomeric, centromeric, and many rRNA and tRNA regions (Figure 1). Analysis of small RNA features showed that these identified small RNAs had a strong preference for 5′ uridine and their size peaked at 20 nt at all three time points (Figure S1A).

We next classified reads based on their match to genomic features into eight pools of small RNAs, namely rRNA, tRNA, small nucleolar RNAs, milRNA (Lee *et al.* 2010b), Dicer-independent small interfering RNA (Lee *et al.* 2010b), masiRNA, mitochondrial RNA, and other unspecified RNAs (Figure S1B). Most 20-nt long RNAs were identified to originate from ribosomal DNA loci (Figure S1C). We noted that the abundance of reads mapping to tRNAs decreased dramatically from 41.5% in PP to 16.9% in 2d PF and then increased slightly to 23.5% in 4d PF. It has been shown that the microRNA-like *milRNA-4* is derived from the precursor of tRNA in vegetative tissue (Yang *et al.* 2013), suggesting that other unknown milRNA genes may account for the high percentage of tRNA in the PP sample. The masiRNA class had a relative large variance of abundance in three time points, ranging from low frequencies of 0.1% at PP and 0.2% at 2d PF to 10-fold greater frequency of 1.9% at 4d PF. This class of small RNAs, with sizes peaking at 25 nt, was primarily derived from 31 loci unique to FGSC 2489 (Table S2). This class also showed preference for a 5′ uridine (Figure S2). This is expected for small RNAs processed by Argonaute-like proteins (Mi *et al.* 2008). In addition, the observed size of 25 nt for masiRNAs is identical to the size of small RNAs generated *in vitro* by *N. crassa* DCL-1, which has been shown to be required for meiotic silencing (Catalanotto *et al.* 2004; Macrae *et al.* 2006; Alexander *et al.* 2008), suggesting that masiRNAs are DCL-1 products. No strand preference for the production of these small RNAs was observed (Table S2), suggesting that the formation of double-stranded RNA (dsRNA) is required.

### Unpaired regions that may undergo meiotic silencing

Analysis of the genomes of FGSC 2489 and FGSC 8820 was performed to identify regions unique to FGSC 2489 that could potentially form unpaired loops and trigger meiotic silencing. Previous work has shown that unpaired regions larger than 700 bp are needed to efficiently trigger meiotic silencing (Lee *et al.* 2004). To identify candidate loci targeted by meiotic silencing we applied the following filters: (1) the region is unique to strain FGSC 2489; (2) the size of unpaired region is larger than 700 bp; (3) small RNAs are only enriched at 4d PF; (4) the ratio of small RNA production to the size of the unpaired region is greater than 1. There were 31 regions that met all criteria. Twenty-four of these contain predicted genes, all of which encode “hypothetical proteins”. The regions ranged in size from 1 kb to 15 kb with 19 smaller than 5 kb (59%), nine between 5 kb and 10 kb, and four longer than 10 kb. The masiRNAs were primarily derived from these 31 unique regions and share similar properties: they are 24∼26 bp long with a peak frequency at 25 bp, they show no specific strand bias, and enrichment for RNAs with a 5′ uridine (Figure S2 and Table S2). Similarity analysis using a curated set of repetitive elements found eight of the regions contained the repeated sequences or remnants of TEs (Table S2).

### Identification of a novel DNA transposon

**Figure 2:**
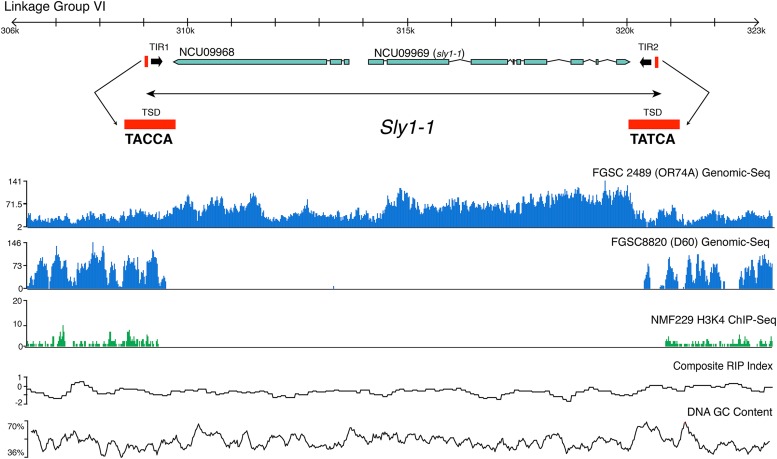
Organization of the *Sly1-1* locus. Schematic showing position and sequences of terminal inverted repeats (TIRs) and target-site duplications (TSD) of *Sly1-1*, and resequencing data from the reference strain FGSC 2489 (*mat A*) and strain FGSC8820 (*mat a*), the partner in the wild-type sexual cross. Chromatin immunoprecipitation sequencing (ChIP-seq) of NMF229 shows absence polymorphism of the *Sly1-1* locus. Repeat-induced point mutation (RIP) and DNA GC% plots indicate no A+T nucleotide skew that would be observed in a region mutated by RIP. The FGSC 2489 resequencing data indicate increased coverage in *Sly1-1* relative to the flanking genomic region, suggesting multiple copies of the element. Clear and almost identical boundaries can be observed where *Sly1-1* is missing in the H3K4me2 ChIP-seq of strain NMF229 and genome sequence of FGSC 8820. We identified genomic sequence reads from FGSC 8820 that perfectly spanned the *Sly1-1* insertion present in FGSC 2489.

**Figure 3:**
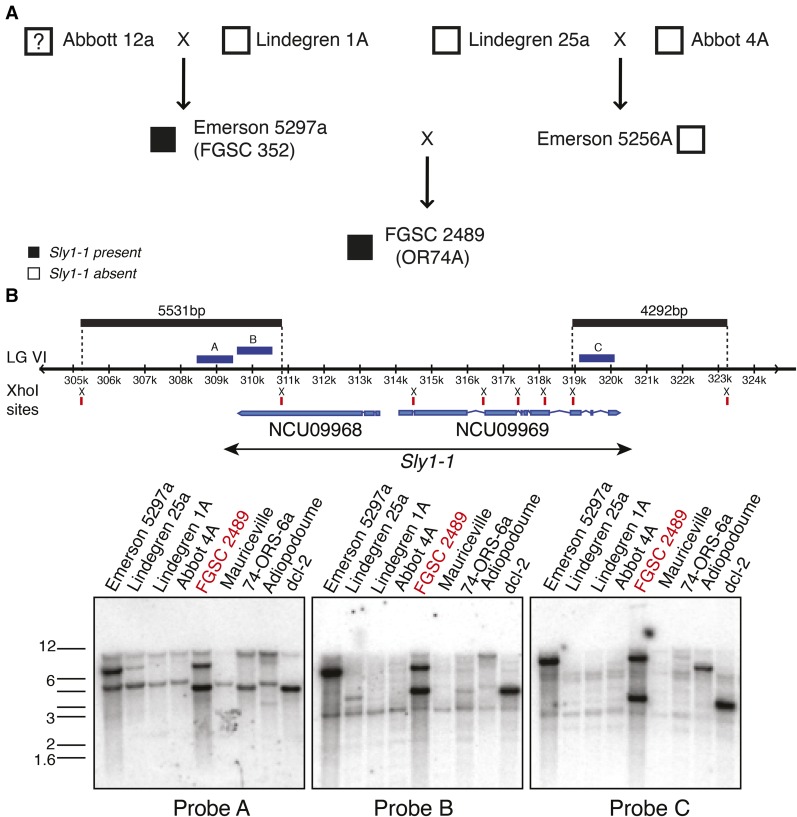
Mapping the presence of *Sly1-1* in the pedigree of FGSC 2489 and related strains. A cross of Lindegren 1A and the presumed Abbott 12a strain (see Newmeyer *et al.* 1987) yielded Emerson 5297a. One copy of *Sly1-1* is present in Emerson 5297a as shown by Southern blot analysis; it remains unknown if *Sly1-1* was introduced from Abbott 12a as the strain may be lost. Lindegren 25a and Abbott 4A produced Emerson 5256A and neither of them carries *Sly1-1*. FGSC 2489 was derived via several backcrosses from a cross between Emerson 5297a and Emerson 5256a. Two copies of *Sly1-1*, instead of just one in Emerson 5297a, are present in the genome of FGSC 2489, as shown in the Southern blots, indicating that *Sly1-1* is potentially active and may have transposed at least once. The FGSC 2489 lineage resulted from multiple back-crosses to Emerson parents, indicated by the dotted line. Genomic DNAs were digested by the restriction endonuclease *Xho*I for Southern blotting analysis. Digested DNA fragments were hybridized with three probes whose positions are illustrated: probe A and probe B hybridized with the 5531-bp fragment of *Sly1-1* at the 5′ end; probe C hybridized with the 4292-bp fragment of *Sly1-1* at the 3′ end.

To analyze endogenous masiRNA in more detail, we focused our analyses on a single 10-kb region unique to FGSC 2489 (Table S2). This region, located on linkage group (LG) VI (309,012 – 320,545 nt), had a high abundance of small RNAs and contained two hypothetical genes, NCU09968 and NCU09969. We noticed that the read coverage of genomic sequencing of this locus in FGSC 2489 was greater than the flanking regions, which indicated that multiple copies of this region were present in the reference genome (Figure 2). Inspection, first by PCR and later by analysis of available genomic sequencing and Southern blots of *N. crassa* strains (Figure 3), indicated that this region was present in the genome of only a few laboratory wild-type strains (FGSC 352), and none of the resequenced laboratory strains (McCluskey *et al.* 2011). This difference was initially noticed in comparison of chromatin immunoprecipitation sequencing samples of strain NMF229 (Smith *et al.* 2011) to the reference genome of the lab wild-type strain, FGSC 2489 (Galagan *et al.* 2003). We named this putative transposon *Sly1-1* and this new DNA transposon family *Sly*, for *Silently*.

**Figure 4:**
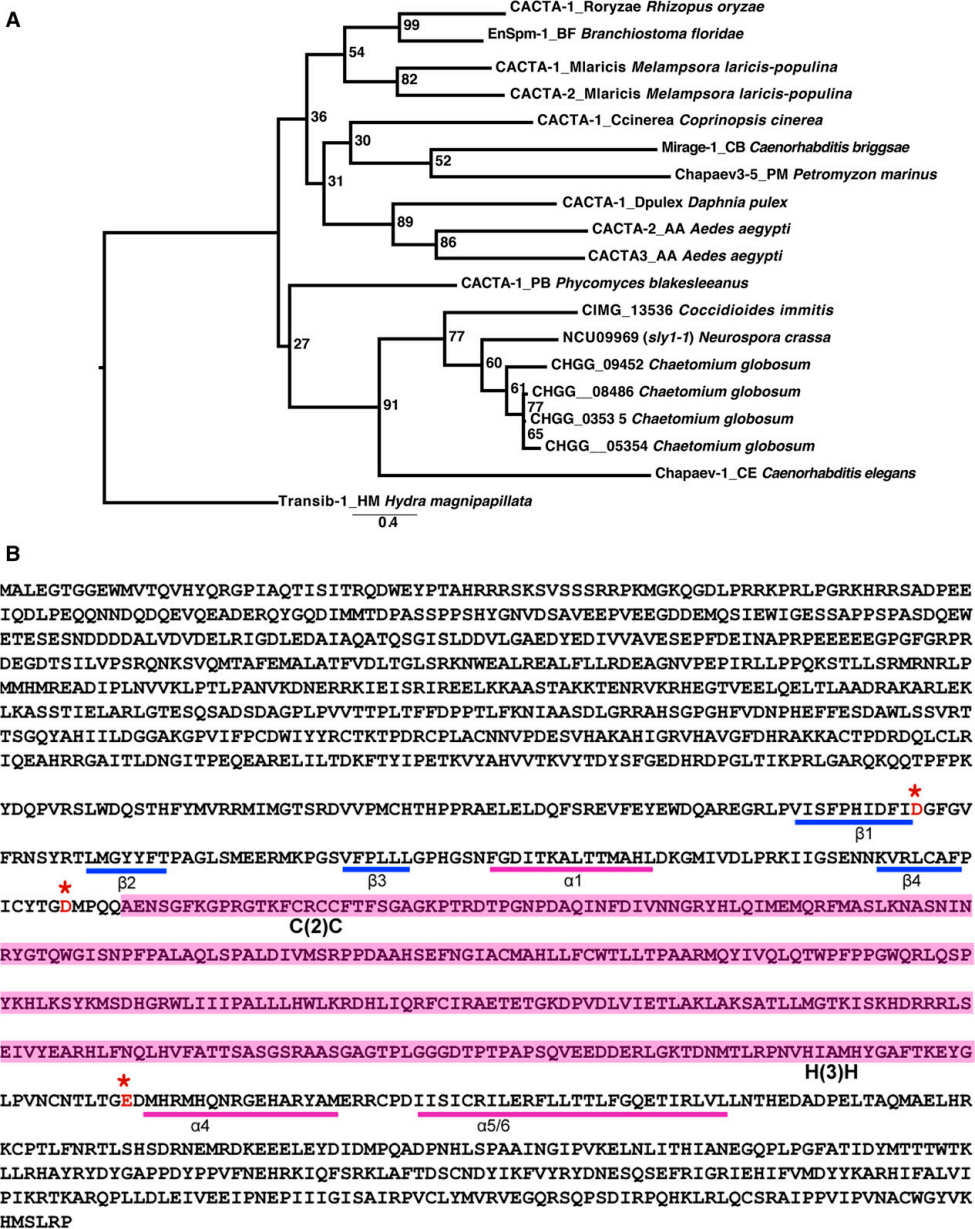
Phylogenetic tree of CMC family and DDE motif present in *sly1-1*. (A) A maximum likelihood phylogenetic tree of CMC family transposases homologous to *sly1-1*. Copies originate from animals and fungi with the species names indicated in the name. (B) Identification of the DDE motif based on expected motif patterns following Yuan and Wessler (2011). The figure shows locations of the beta sheets (β) or alpha helices (α) protein folds, the presence of Cysteine-XX-Cysteine [C(2)C] and Histidine-XXX-Histidine [H(3)H] motifs for the CMC family, and the location of the acidic residues adjacent to the predicted protein folds. The area highlighted in pink between the D and E contains multiple alpha helices.

The original locus on LG VI contains a complete copy of *Sly1-1*. The region is 11,534 bp long and includes an intact transposase domain-containing gene, NCU09969, named *sly1-1*, and a gene of unknown function, NCU09968, which has similarity to *Chaetomium globosum* CHGG_09452 (Figure 2). Phylogenetic analysis of NCU09969 identified it as member of the CMC (CACTA-Mirage-Chapaev) superfamily of DNA transposons (Figure 4A), based on conservation of DDE motifs found in the transposase domain and other conserved residues (Figure 4B) (Yuan and Wessler 2011). The arrangement of genes within *Sly1-1* is conserved in other species. Homologs of both ORFs were found as adjacent and divergently transcribed genes in *Ch. globosum* (CHGG_09451 and CHGG_09452) while in *Coccidioides immitis* only the *Sly* transposase could be identified as gene (CIMG_13536).

**Figure 5:**
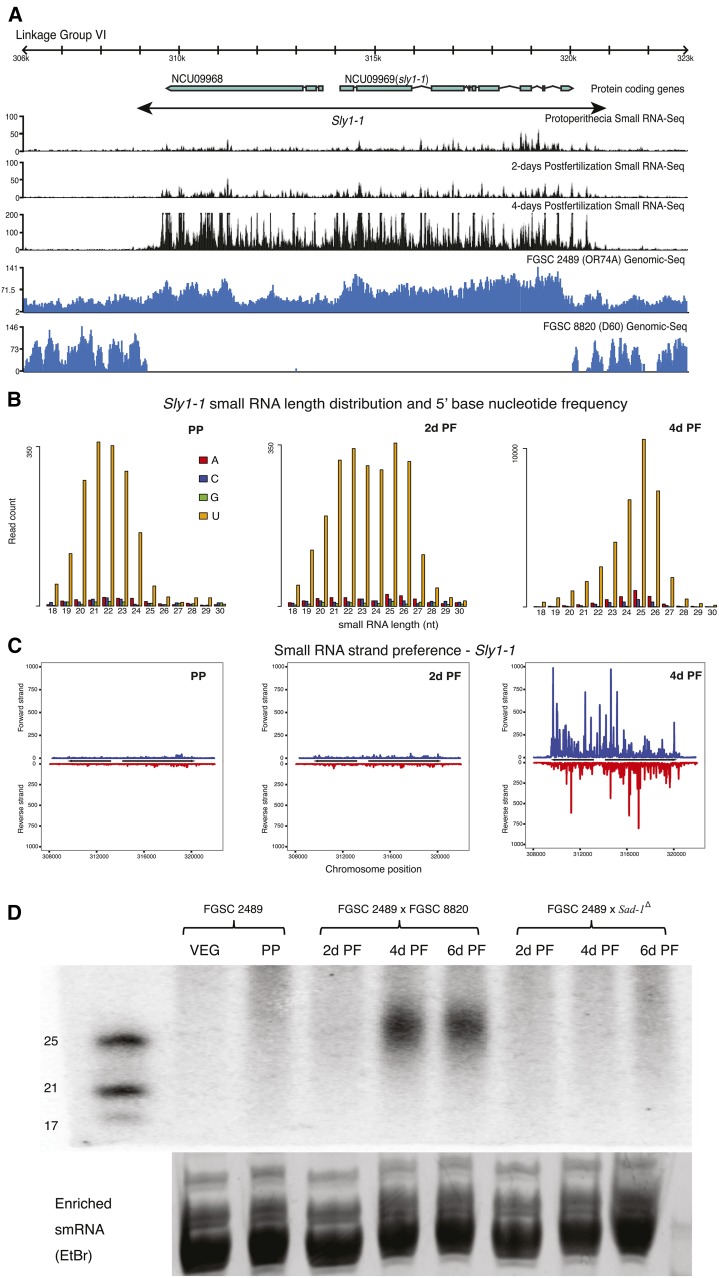
Illustration and analysis of small RNAs from *Sly1-1*. (A) Small RNA sequence profiles from three sexual developmental time points are aligned to genomic sequencing reads from FGSC 2489 and FGSC 8820 and a diagram to show the structure of *Sly1-1*. Small RNA production precisely matched the boundaries of *Sly1-1*. (B) Length distribution and 5′ base nucleotide frequency of small RNAs produced from *Sly1-1* at the same three time points as above [protoperithecial (PP); 2 d postfertilization (PF) 2d PF and 4d PF]. Most of the small RNAs at 4d PF are 25 nt long and mostly have uridine as 5′ base. (C) There is no obvious strand preference of small RNA from *Sly1-1* at the same three time points. Few small RNAs are produced at PP and 2d PF; abundant small RNAs are produced at 4d PF. (D) Small RNA northern blot of small RNAs from a cross (FGSC 2489 × FGSC 8820) in which meiotic silencing should occur and a cross (FGSC 2489 × *Sad-1^Δ^*) in which meiotic silencing should be absent. Strong signals of small RNA (25 nt long) were detected at 4d PF and 6d PF in the wild-type cross but not in the mutant cross that lacks SAD-1.

TIRs and TSDs are hallmarks of DNA transposons (Potter *et al.* 1980). We identified two TIRs (TIR1 and TIR2) and TSDs (Figure 2) at the boundary of *Sly1-1* by comparing *Sly1-1* with non-*Sly1-1*−containing strains (FGSC8820; FGSC7022; FGSC1363; FGSC106; D48, FGSC8088; D106, FGSC8866; NMF229). The comparison between strains with no *Sly1-1* and FGSC 2489 helped define the precise boundary of the transposon and the locations of the TIRs and TSDs and we observe that small RNA read abundance drops off sharply outside of the delineated locus (Figure 5A). The sequences of two TSD located at the boundaries are not exactly the same, likely due to RIP as the third nucleotide of TSD1, cytosine, is replaced by thymine.

To survey the presence of *Sly1-1* in *N. crassa* laboratory strains we carried out Southern blotting, which revealed that *Sly1-1* is not common and is present at low copy number (Figure 4B and Figure S3). Only strains in the FGSC 2489 lineage (Gavric and Griffiths 2004; Newmeyer *et al.* 1987), including FGSC 352 (Emerson 5279a) and strains derived from crosses with FGSC 2489, such as the collection of single-gene deletion strains (Colot *et al.* 2006), contain at least two copies of *Sly1-1* (Figure 4B and Figure S3). Our survey of all available re-sequencing data of *N. crassa* strains (McCluskey *et al.* 2011), including the Mauriceville strain (Pomraning 2012), *N. tetrasperma* (Ellison *et al.* 2011), *N. discreta* (a homothallic *Neurospora* species) (Gioti *et al.* 2013), and *Sordaria macrospora* (Nowrousian *et al.* 2010), suggests that *Sly1-1* is largely absent from the tested strains (data not shown); inspection of unreleased genome data from the pedigree of FGSC 2489 showed presence of *Sly1-1* only in FGSC4200, FGSC9718, and FGSC987 (S. Baker, K. McCluskey, I. Grigoriev, and J. Stajich, unpublished results). The absence of *Sly1-1* in many of these strains suggests that it arrived or was activated only recently in *N. crassa*.

We used BLAST (Altschul *et al.* 1997) to search the FGSC 2489 genome with the *Sly1-1* sequence to identify the additional copy inferred by the second band in the Southern blots. A partial copy, which we named *Sly1-2*, is present in assembly 12 within centromeric DNA on LG II (1,151,460–1,155,073 nt). In FGSC 2489 we found no evidence for RIP in the two *Sly* copies. The second copy may represent evidence of recent transposition. However, confidence in the assembly of the genome in this region is not high as repetitive regions are a hallmark of centromeric DNA and difficult to assemble. Because probes for both ends of Sly1-1 in the Southern blot showed evidence for two copies it is likely *Sly1-2* is intact, just not assembled correctly (Figure 3B). The original and transposed positions of *Sly1-1* are similar in that both show cytosine DNA methylation in the *Sly1-1* flanks, detected by high-throughput sequencing of immunopurified methylated DNA (Smith *et al.* 2011). Both regions also show absence of the centromere-specific H3, CenH3 and histone H3 lysine 9 trimethylation (H3K9me3) but presence of H3K4me2 (Smith *et al.* 2011). The identification of two, nearly identical stretches of DNA in the *N. crassa* genome that have not been RIPed is unexpected as previous work had identified only few regions with predicted genes that showed high similarity (Galagan *et al.* 2003; Smith *et al.* 2011).

Searching the FGSC 2489 reference genome with TIR1 and TIR2 of *Sly1-1* showed that *Sly1-2* contained the *Sly1-1* TIR1 but lacked TIR2 (Figure S4A). We also detected two additional loci that contained nearly identical TIR pairs, located on LG V and LG I; these were named *Sly1-3* and *Sly1-4*, respectively (Figure S4A and Table S3). *Sly1-3* and *Sly1-4* are both absent in the genome of FGSC 8820. *Sly1-2* is only about 3614 bp long, 100% identical to *Sly1-1*, has the same TSD as *Sly1-1* adjacent to the sequence of TIR1 and contains a putative gene, NCU16528. The 3′ end of *Sly1-2* is not completely assembled and stretches across two contigs. It is possible that *Sly1-2* is larger in size and that its TIR2 is located in an unsequenced region. The unequal read coverage observed for the two loci in *Sly1-1* (Figure 2) may arise because the total reads aligned are titrated between two chromosome locations for NCU09968 (*e.g.*, *Sly1-1* and *Sly1-2*) while only one NCU09969 sequence is in the reference assembly at *Sly1-1* which results in higher computed read coverage. *Sly1-3* and *Sly1-4* are very similar to each other: BLASTN showed query coverage ∼95% with identity ∼85% (E value < 0.001), both are ∼12 kb long, contain no identified open reading frames, and have nearly the same TSDs adjacent to TIRs. RIPCAL (Hane and Oliver 2008) indicates that *Sly1-3* and *Sly1-4* have undergone RIP. This situation is consistent with the observation that alignments of *Sly1-3* and *Sly1-4* to *Sly1-1* cover 94% and 91% of the sequences, but only have 67% and 72% identity, respectively.

BLASTX searches with *Sly1-2* identify significant similarity to NCU09968 (query coverage = 61%; E value = 0 and identity = 99%). The translated search of *Sly1-3* and *Sly1-4* also reveal similarity to both NCU09968 (*Sly1-3*: query coverage = 29%; E value < 0.001; identity = 54%; *Sly1-4*: query coverage = 30%; E value < 0.001; identity = 60%) and NCU09969 (*Sly1-3*: query coverage = 27%; E value < 0.001; identity = 53%; *Sly1-4*: query coverage = 28%; E value < 0.001; identity = 54%), respectively. Summarizing all of these results, we conclude that *Sly1-1* is a recently active TE, with at least two inactivated copies that underwent RIP (*Sly1-3* and *Sly1-4*), but a partial copy which is identical in sequence and thus not mutated by RIP, *Sly1-2*, that may be active. Detection of similar but mutated copies of both genes, NCU09968 and *sly1-1*, in *Sly1-3* and *Sly1-4* suggests that the entire *Sly1-1* locus transposed and not just the transposase, *sly1-1*.

### Meiotic silencing machinery is required for the production of *Sly1-1* small RNAs

To characterize transcription and small RNAs produced from *Sly1-1* as a consequence of meiotic silencing, we first determined when the *sly1-1* transposase gene (NCU09969) was expressed. Analysis of published transcriptome data (Wang *et al.* 2012) detected no *sly1-1* transcript during vegetative growth. However, during the sexual cycle, gene expression was detected at 0, 2, 24, 48, 72, 96, 120, and 144 hr after crossing FGSC 2489 with FGSC 4200. The RPKM (reads per kilobase per million mapped) values for *sly1-1* from RNA-seq ranged from 6 to 10 (Wang *et al.* 2014), indicating that it is expressed during sexual development, if not at a high level.

We also examined the small RNAs produced from *Sly1-1* during later stages of sexual development. Karyogamy occurs in perithecia 3−4 d after fertilization (Raju 1980) and meiotic silencing is first detected at karyogamy (Shiu *et al.* 2001). Our small RNA profiles support these previous results but show small RNA production occurring at the same time as karyogamy, not after, which is when MSUD is thought to occur (Shiu *et al.* 2001). This difference may reflect a delay between when small RNAs are produced, the process of aberrant RNA recognition, and when silencing machinery acts on transcripts. We observed an abundance of small RNAs produced from *Sly1-1* at 4d PF, compared with samples collected at PP stages or 2d PF (Figure 5, C and D). Similar to the small RNAs described previously and to those identified to be from the *Roundspore* (*Rsp*) locus in an unpaired and MSUD inducing cross (Hammond *et al.* 2013a), the small RNAs from *Sly1-1* have features typical of those produced during quelling (Figure 5, B and C and Table S2). The size and expression pattern of *Sly1-1* derived small RNAs were also confirmed by small RNA northern blots (Figure 5D). Samples from PP, 2d PF, 4d PF, and 6d PF were examined and small RNAs were detected at 4d and 6d PF, indicating that the production of small RNAs was sustained.

Previous work showed that *sad-1* is essential for meiotic silencing and is specifically expressed during sexual development (Shiu *et al.* 2001, 2006). SAD-1 is homologous to the RNA-dependent RNA polymerase, QDE-1. As noted previously, QDE-1 is involved in quelling (Romano and Macino 1992; Cogoni and Macino 1997; Fulci and Macino 2007), whereas SAD-1 operates during meiosis, where it is thought to convert single-stranded aberrant RNA transcribed from unpaired regions into dsRNA, producing the substrate that is cleaved by DCL-1 to generate small interference RNA (Kelly and Aramayo 2007; Aramayo and Pratt 2010; Aramayo and Selker 2013). To test whether the small RNA production observed from this unpaired region was dependent on SAD-1, we crossed the dominant mutant allele *Sad-1^Δ^* (present in a genetic background lacking *Sly1-1*; FGSC 8740), to FGSC 2489. RNA collected at 2d, 4d, and 6d PF was tested by northern analyses. No small RNA from the *Sly1-1* locus was detected under these conditions (Figure 5D). This result suggested a relationship between synthesis of dsRNA and small RNA production, both dependent on SAD-1.

We also tested whether the production of unprocessed *sly1-1* RNAs was dependent on SAD-1. We used a method developed to detect qiRNA (Lee *et al.* 2009) and examined transcript levels from the intergenic regions outside of the *sly1-1* gene (NCU09969) to test for aberrant RNA production. Quantitative PCR showed that transcripts originating from downstream regions of the *sly1-1* locus were highly induced from 2d PF to 6d PF (Figure S5). This observation suggested that RNA is required to initiate and maintain the production of aberrant and, presumably, small RNAs. In the cross between *Sad-1^Δ^* and FGSC 2489, transcripts accumulated at a high level at 2d PF, but decreased dramatically at 4d PF and 6d PF (Figure S5). This decrease in transcript levels indicated that the lack of SAD-1 did not block the production of aberrant, or any, RNA initially, but may have stalled dsRNA production, which resulted in the suppression of the production of small RNAs. The exact mechanisms underlying this surprising finding will need to be addressed by future experiments.

In summary, our study provides evidence for a novel and apparently intact TE in the widely used laboratory wild-type strain of *N. crassa*, a genome thought to be lacking active transposons (Selker *et al.* 2003). Based on Southern blots examining the pedigree and genome of FGSC 2489, and further validated by examination of whole genome sequences of most of the strains in this pedigree, it appears that *Sly1-1* was acquired recently and has actively transposed, with evidence for at least two intact copies. Examination of additional wild strains from ongoing resequencing efforts (S. Baker, K. McCluskey, I. Grigoriev, J. Stajich, and unpublished data) provided a better understanding of the origins and timing of acquisition of this newly described selfish genetic element. Our study also provides evidence for the original hypothesis that meiotic silencing targets unpaired DNA created by a transposon insertion and is an effective genome defense mechanism. The unpaired region triggers the production of masiRNA during sexual development, approximately 4−6 d after fertilization, when karyogamy is expected to occur.
